# Mindfulness and self-compassion based intervention program to prevent burnout in medical and dentistry students

**DOI:** 10.1192/j.eurpsy.2021.1228

**Published:** 2021-08-13

**Authors:** F. Carvalho, C. Cabaços, M. Carneiro, A. Araujo, J. Azevedo, M. Marques, A. Manão, A. Macedo, A.T. Pereira

**Affiliations:** 1 Institute Of Psychological Medicine, Faculty Of Medicine, University of Coimbra, coimbra, Portugal; 2 Institute Of Psychological Medicine, Faculty Of Medicine, University of Coimbra, Coimbra, Portugal

**Keywords:** burnout, Medical Students, Mindfulness based intervention, Self-compassion

## Abstract

**Introduction:**

Burnout occurs in every stage of a medical graduation and career. In the first years of graduation, is affects 35-45% of medical and dentistry students. This has severe consequences, such as: higher levels of suicidal ideation, substance abuse, medical errors and medical neglect; lower levels of empathy and self-compassion - essential to the quality of healthcare. Students with certain personality traits (e.g., neuroticism and, particularly, perfectionism) are more vulnerable to emotional dysregulation when facing stressors of daily life. Our recent studies proved that mindfulness and self-compassion can attenuate the effect of perfectionism on psychological distress.

**Objectives:**

To present the rational, materials, methodology and preliminary results of our project COMBURNOUT, aimed to develop, implement and assess the efficacy of a mindfulness and self-compassion-based intervention to prevent burnout in medical and dentistry students.

**Methods:**

Students with high levels of burnout, psychological distress and perfectionism will be randomly assigned to intervention (8 weekly sessions) or control groups. The sessions will be composed by psychoeducation about burnout, mindfulness and self-compassion practices, within and between sessions. The follow up will include three assessment moments until a year after the intervention.

**Results:**

We expect that the experimental group will present significantly lower levels of burnout, psychological distress and perfectionism, and higher levels of emotional regulation skills.

**Conclusions:**

The facilitators training and the manualization are guaranties of standardization and sustainability. If the positive impact of COMBURNOUT is verified, we intend to provide the program to medical/dentistry students from all over the country.

